# BIRC5 Inhibition Is Associated with Pyroptotic Cell Death via Caspase3-GSDME Pathway in Lung Adenocarcinoma Cells

**DOI:** 10.3390/ijms241914663

**Published:** 2023-09-28

**Authors:** Qingwei Zhang, Ximing Chen, Yingying Hu, Tong Zhou, Menghan Du, Run Xu, Yongchao Chen, Pingping Tang, Zhouxiu Chen, Jiamin Li

**Affiliations:** 1 Department of Pharmacology (State-Province Key Laboratories of Biomedicine-Pharmaceutics of China, Key Laboratory of Cardiovascular Medicine Research, Ministry of Education) at College of Pharmacy, Harbin Medical University, Harbin 150081, China; 2 NHC Key Laboratory of Molecular Probe and Targeted Diagnosis and Therapy, Molecular Imaging Research Center (MIRC) of Harbin Medical University, Harbin 150081, China

**Keywords:** lung adenocarcinoma, apoptosis, pyroptosis, DNA methylation, BIRC5

## Abstract

Lung adenocarcinoma (LUAD) is a prevalent type of thoracic cancer with a poor prognosis and high mortality rate. However, the exact pathogenesis of this cancer is still not fully understood. One potential factor that can contribute to the development of lung adenocarcinoma is DNA methylation, which can cause changes in chromosome structure and potentially lead to the formation of tumors. The baculoviral IAP repeat containing the 5 (BIRC5) gene encodes the Survivin protein, which is a multifunctional gene involved in cell proliferation, migration, and invasion of tumor cells. This gene is elevated in various solid tumors, but its specific role and mechanism in lung adenocarcinoma are not well-known. To identify the potential biomarkers associated with lung adenocarcinoma, we screened the methylation-regulated differentially expressed genes (MeDEGs) of LUAD via bioinformatics analysis. Gene ontology (GO) process and the Kyoto Encyclopedia of Genes and Genomes (KEGG) were applied to investigate the biological function and pathway of MeDEGs. A protein–protein interaction (PPI) network was employed to explore the key module and screen hub genes. We screened out eight hub genes whose products are aberrantly expressed, and whose DNA methylation modification level is significantly changed in lung adenocarcinoma. BIRC5 is a bona fide marker which was remarkably up-regulated in tumor tissues. Flow cytometry analysis, lactate dehydrogenase release (LDH) assay and Micro-PET imaging were performed in A549 cells and a mouse xenograft tumor to explore the function of BIRC5 in cell death of lung adenocarcinoma. We found that BIRC5 was up-regulated and related to a high mortality rate in lung adenocarcinoma patients. Mechanically, the knockdown of BIRC5 inhibited the proliferation of A549 cells and induced pyroptosis via caspase3/GSDME signaling. Our findings have unraveled that BIRC5 holds promise as a novel biomarker and therapeutic target for lung adenocarcinoma. Additionally, we have discovered a novel pathway in which BIRC5 inhibition can induce pyroptosis through the caspase3-GSDME pathway in lung adenocarcinoma cells.

## 1. Introduction

Lung cancer has emerged as the leading cause of cancer-related deaths globally [1,2]. The primary histological subtype of lung cancer, known as lung adenocarcinoma (LUAD), encompasses a spectrum of preinvasive lesions to invasive adenocarcinoma [3,4,5]. This subtype is associated with higher mortality rates, primarily due to limited understanding of the molecular mechanisms underlying cancer development. Therefore, it is crucial to explore novel biomarkers and uncover the precise molecular targets of LUAD. Differentially expressed genes (DEGs) are identified as the result of genetic defects (e.g., EGFR and TP53 mutation) and epigenetic modifications (such as DNA methylation and RNA methylation), which play a vital role in promoting tumorigenesis, progression and metastasis of LUAD [6,7,8]. 

With the continuous improvement of DNA microarrays and genechip technology, high throughput sequencing technology is now considered a promising tool for identifying epigenetic changes in cancer-related genes. This has led to the generation of a large number of open-access datasets in publicly accessible database repositories. The most widely used public database in the field of oncology are the Gene Expression Omnibus (GEO) and The Cancer Genome Atlas (TCGA) which contain thousands of clinical information and gene sequencing data. The database can provide a well-rounded analysis of aberrant gene expression in various cancers [7,9]. Lots of DEGs associated with LUAD were unequivocally identified by some gene expression profiling studies, based on reanalyzed gene expression data derived from different institutes. Usually, the analysis of aberrant gene expression can provide valuable information for the objective identification of novel biomarkers for LUAD. However, the traditional analysis of DEGs from a single study has its limitations. By obtaining overlapping genes from multiple available datasets and conducting comprehensive bioinformatics analysis, more representative and accurate results can be obtained.

DNA methylation is a common mechanism of epigenetic regulation that affects gene expression and various cellular processes. Several studies have demonstrated a close relationship between DNA methylation and the progression of cancer [10,11,12,13]. However, a comprehensive profile that includes both DEGs and DNA methylation in LUAD is still lacking. In this study, we analyzed the gene expression microarray dataset (GSE118370) and DNA methylation profiling microarray dataset (GSE139032) from GEO database. The methylation-regulated differentially expressed genes (MeDEGs) between human LUAD tissue and healthy lung tissue were identified from the previous two microarray datasets. Subsequently, gene-related functional enrichment was performed using gene ontology (GO) and the Kyoto Encyclopedia of Genes and Genomes (KEGG) databases, and the protein–protein interaction (PPI) network construction was constructed to screen highly cancer-related genes for LUAD [14]. Then, we identified eight hub genes, with the baculoviral IAP repeat containing the 5 (BIRC5) gene, which is considered a key biomarker. We further validated its regulatory effect on cell death in LUAD.

The BIRC5, also known as Survivin, was a member of the inhibitor of the apoptosis family. Survivin is tumor-specific gene, expressed only in tumors and embryonic tissues, and is closely related to differentiation, proliferation, invasion and metastasis of tumor cells [15,16,17]. In our research, we observed an association between higher BIRC5 expression and unfavorable prognosis in LUAD. However, differing from the traditional view, our results show that the BIRC5 inhibition can mediate apoptosis to pyroptosis via caspase3/GSDME in LUAD; these findings provide a key biomarker and explore new therapeutic targets for lung adenocarcinoma diagnosis and treatment. 

## 2. Results

### 2.1. Identification of MeDEGs in Lung Adenocarcinoma

**Figure 1:**
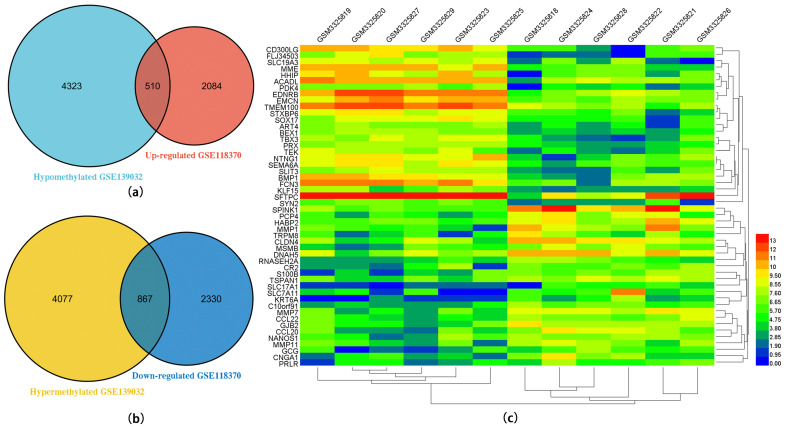
Venn diagram of methylation-regulated differentially expressed genes (MeDEGs) from GSE118370 and GSE139032 datasets and of the top 50 MeDEGs shown on the heatmap in tumor and peri-tumor. (**a**) Venn diagram of hypomethylated and upregulated genes. (**b**) Venn diagram of hypermethylated and downregulated genes. (**c**) Heat map of the top 50 MeDEGs.

GEO2R is a data analysis tool of GEO datasets that was used for further screening of DEGs from the expression profile microarray database (GSE118370), and DMGs from the DNA methylation microarray database (GSE139032). As a result, A total of 5791 DEGs were obtained following GSE118370 data processing. Compared to the adjacent normal ones, 2594 genes were markedly increased, and 3197 genes were observably decreased in the tumor. Moreover, 4944 hypermethylated genes and 4833 hypomethylated genes from GSE139032 were screened. We identified 510 upregulated and hypomethylated genes and 867 downregulated and hypermethylated genes by overlapping DEGs and DMGs from two GSE datasets (Figure 1a,b). The top 25 upregulated and hypomethylated genes and 25 downregulated and hypermethylated genes according to the fold change (FC) value for heat map analysis were recorded (*p* < 0.05) (Figure 1c).

### 2.2. Functional Enrichment Analysis of MeDEGs

To explore the main function of MeDEGs, enrichment analyses were performed using FunRich. The results of GO analysis revealed that the upregulated and hypomethylated genes were related to “oxidative stress and tumor necrosis factor”, “active transmembrane transporter activity”, “cysteine-type endopeptidase inhibitor activity” and “apical part of cell, cell-cell junction”. Conversely, the downregulated and hypermethylated genes were primarily associated with “ameboidal-type cell migration”, “DNA-binding transcription activator activity” and “focal adhesion” (Figure S1a–f). The top three KEGG pathways enriched in upregulated and hypomethylated genes included the tight junction, cell adhesion molecules and glycosphingolipid biosynthesis-lacto and neolacto series (Figure S1g). For the downregulated and hypermethylated genes, the most significant results comprised the MAPK signaling pathway, vascular smooth muscle contraction, focal adhesion, calcium signaling pathway, cGMP-PKG signaling pathway, axon guidance, cell adhesion molecules, dilated cardiomyopathy, hypertrophic cardiomyopathy, morphine addiction and renin secretion (Figure S1h). 

### 2.3. PPI Network Construction

**Figure 2:**
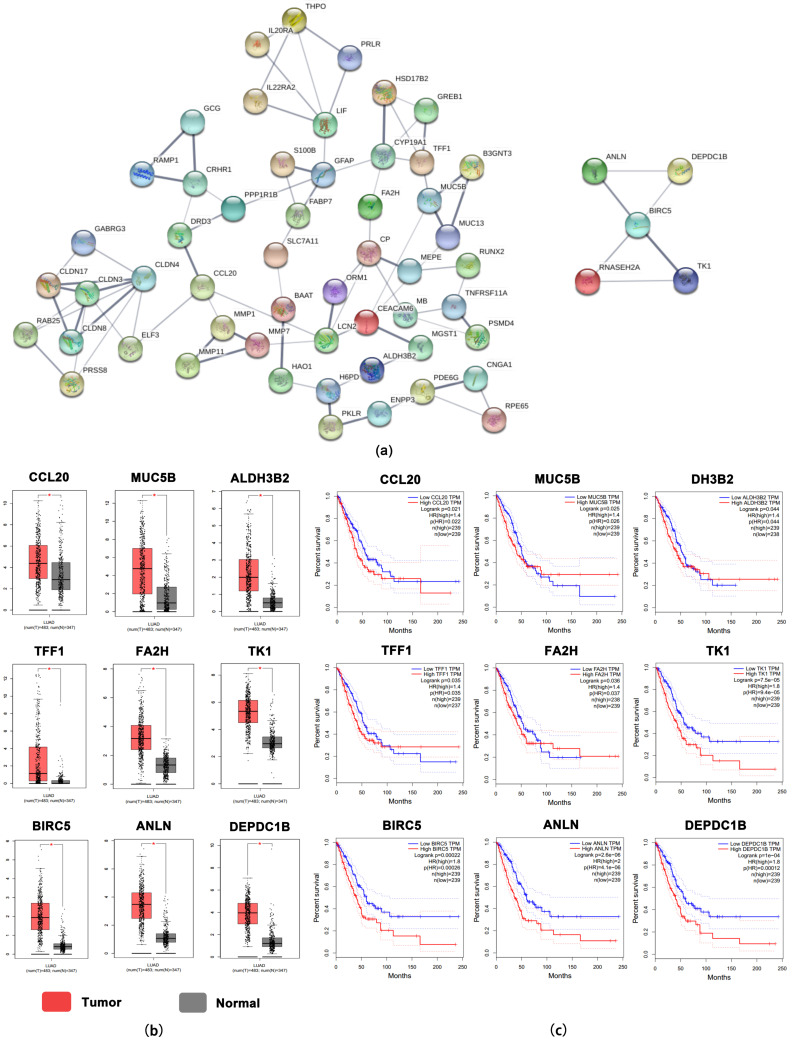
Protein–protein interaction (PPI) network and the expression validation and survival analysis of upregulated and hypomethylated genes. (**a**) The PPI network analysis of the 58 upregulated and hypomethylated genes, visualized with Cytoscape tool. (**b**) The distribution of nine upregulated and hypomethylated genes expression value in the TCGA LUAD dataset. Values of * *p* < 0.05 vs. Normal (**c**) Kaplan–Meier overall survival curves were performed for survival analysis of upregulated and hypomethylated genes.

To establish the protein–protein triple regulatory network and explore the interaction between the proteins encoded by MeDEGs, the PPI network of upregulated and hypomethylated expression genes and downregulated and hypermethylated expression genes was predicted and structured with the STRING database. An interaction with a combined score greater than 0.4 and a *p* value less than 0.05 were defined as statistically significant. The total PPI network validation and hub gene validation were performed with the Cytoscape software, two modules for hypomethylated high-expression genes (Figure 2a) and three modules for hypermethylated low-expression genes (Figure S2a) were screened in the PPI network with the standard of combined scores being greater than three, and nodes being greater than five. Finally, 100 hub genes were identified using CytoHubba with a degree of ≥10, with 58 genes which were defined as upregulated and hypomethylated and 42 genes which were downregulated and hypermethylated (Supplementary File S1). 

### 2.4. Verification of Hub Genes and Selection of a Model with LUAD-Specific Prognostic Value 

We next performed multiple validation on the screened hub genes. GEPIA is a newly developed interactive web server for analyzing the RNA sequencing expression data of 9736 tumors and 8587 normal samples from The Cancer Genome Atlas (TCGA) and the Genotype-Tissue Expression (GTEx) Project, using a standard processing pipeline. To further assess the identified hub genes, the expression of these hub genes in LUAD tissues and overall survival for the hub genes were analyzed in the TCGA database with GEPIA. As a result, compared to adjacent lung tissues, we screened nine upregulated and hypomethylated hub genes (CCL20, MUC5B, ALDH3B2, TFF1, FA2H, TK1, BIRC5, ANLN and DEPDC1B) in lung adenocarcinoma which were significantly elevated, and the high expression of these genes was significantly associated with poor overall survival in patients with LUAD (Figure 2b,c). Furthermore, four downregulated and hypermethylated genes (ADRB2, ENG, PECAM1 and SLIT3) identified from GSE database were found to be downregulated in LUAD and the downregulation of ADRB2, ENG, PECAM1 and SLIT3 were associated with the worse overall survival in LUAD patients (Figure S2b,c). The DNA methylation data of all 13 hub genes were validated using DiseaseMeth and the results were listed in Table S1. The above results showed that the methylation levels and expression of six upregulated and hypomethylated hub genes (CCL20, MUC5B, ALDH3B2, TFF1, FA2H and BIRC5) and two downregulated and hypermethylated genes (SLIT3 and ADRB2) were consistent with the screen results of databases (GSE139032 and GSE118370). Additionally, the expression levels of these genes were significantly associated with LUAD prognosis. 

### 2.5. The Expression of BIRC5 Was Significantly Increased in LUAD 

**Figure 3:**
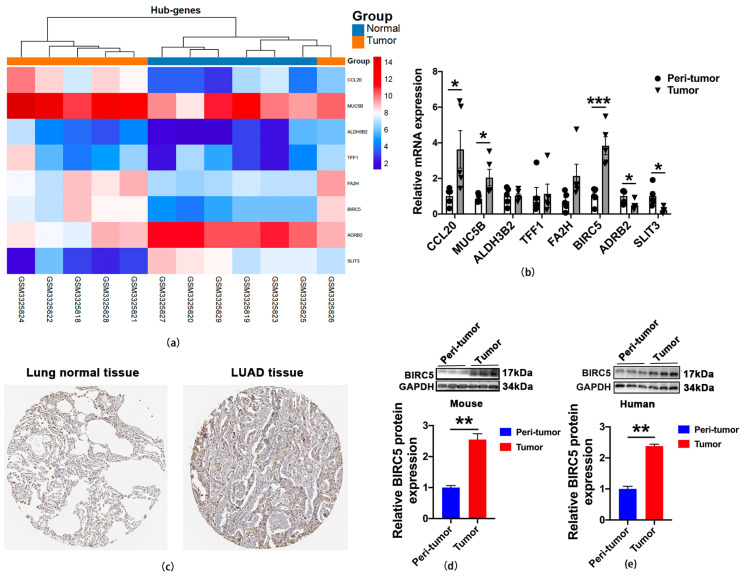
Validation of the key hub genes. (**a**) Heat map of eight hub genes. (**b**) The qRT-PCR analysis of eight candidate genes in human lung adenocarcinoma and peri-tumor samples. Values of * *p* < 0.05 and *** *p* < 0.001 vs. peri-tumor (n = 5). (**c**) Representative immunohistochemistry images of BIRC5 from normal lung tissue and LUAD tissues extracted from the Human Protein Atlas database. (**d**) The protein level of BIRC5 in mouse orthotopic LUAD and adjacent normal tissue, value of ** *p* < 0.01 vs. peri-tumor (n = 6). (**e**) The protein level of BIRC5 in human LUAD and peri-tumor samples, value of ** *p* < 0.01 vs. peri-tumor (n = 3).

We performed a series of validation steps in various bioinformation datasets. Finally, as shown in Figure 3a, we identified eight candidate genes that were regulated epigenetically with DNA methylation and potentially had important roles in LUAD. To further investigate these candidate genes, we conducted qRT-PCR analysis on eight MeDEGs. The results showed that the elevation of BIRC5 was more significant at the mRNA level in the tumor group (Figure 3b). Furthermore, we investigated the relationship between candidate genes and the LUAD tumor stage with GEPIA. The result indicated that the BIRC5 was more positively associated with tumor progression than the other candidate genes (Figure S3a–e). Next, we validated the protein level expression of BIRC5 using the Human Protein Atlas database (https://www.proteinatlas.org/; accessed on 13 September 2023) [18]. The results revealed a noteworthy elevation in the BIRC5 levels in LUAD in comparison to the levels found in normal lung tissue (Figure 3c). Western blot analysis of tumor tissues in an orthotopic tumor model of BALB/c nude mice demonstrated a remarkable upregulation of BIRC5 expression compared to the peri-tumor (Figure 3d). To further explore the expression level of BIRC5 in the patients with LUAD, we collected tumor tissue and paracancer tissue from clinical LUAD patients. Western blot analyses were used to confirm the amount of BIRC5 expression in clinical samples, and BIRC5 protein levels were substantially elevated in tumor tissues as compared to neighboring peri-tumor tissues (Figure 3e). These results provide strong evidence to support our conclusion that BIRC5 is upregulated in LUAD.

### 2.6. Correlation between Expression of BIRC5 and the Immune Infiltration

**Figure 4:**
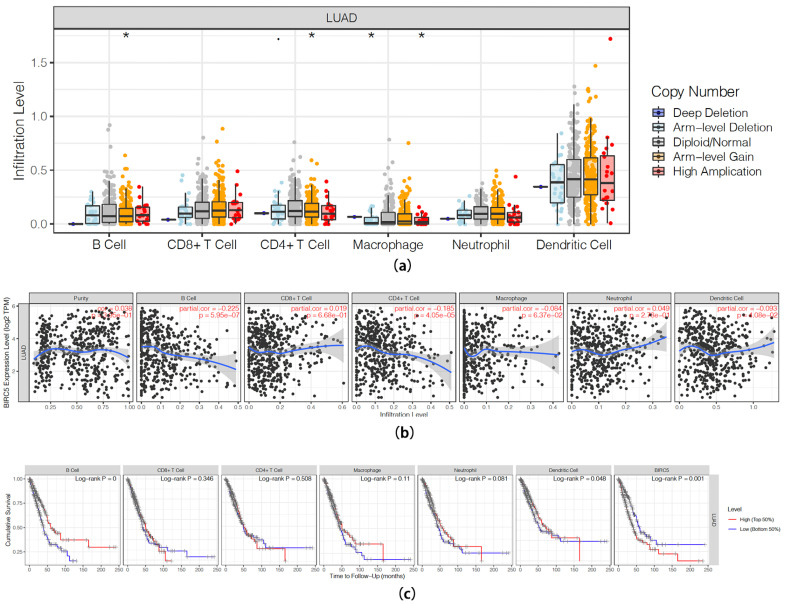
Correlation between expression of BIRC5 and the immune infiltration. (**a**) Correlation of BIRC5 gene copy numbers with the infiltration of various immune cells in LUAD, values of * *p* < 0.05. (**b**) Association between BIRC5 expression and immune cells infiltration level in LUAD, the blue line represents the trend curve. (**c**) Different types of immune cell infiltration survival analysis.

Research has shown that the presence of tumor-infiltrating lymphocytes (TILs) is a significant indicator of both sentinel lymph node (SLN) status and overall survival in certain types of cancer [17]. To investigate whether the association between higher BIRC5 expression and a poorer prognosis in LUAD patients is influenced by the abundance of immune infiltrates in tumor tissues, we performed an immune infiltration analysis using TIMER (https://cistrome.shinyapps.io/timer/; accessed on 9 January 2021). This analysis aimed to assess the potential correlation between BIRC5 expression and immune infiltration levels in LUAD. The SCNA module was utilized to compare tumor infiltration levels among tumors with different somatic copy number alterations for BIRC5. Box plots were used to visually represent the distributions of each immune cell type at each copy number status of BIRC5 in LUAD. The results suggest a slight correlation between altered BIRC5 gene copy numbers and the infiltration of various immune cells, including B cells, CD4+ T cells, macrophages and dendritic cells, which indicated that altered BIRC5 gene levels may affect immune cell infiltration in LUAD (Figure 4a). Further analysis revealed that the expression of BIRC5 was negatively correlated with infiltrating levels of B cells, CD4^+^ T cells and dendritic cells in the tumor microenvironment of LUAD (*p* < 0.05). Among them, the correlation between the infiltrating levels of B cells and the level of BIRC5 is more obvious (correlation = −0.225, *p* < 0.001) (Figure 4b). Finally, to further investigate the impact of immune infiltration on clinical prognosis of LUAD patients, we conducted additional evaluations on the association between immune infiltration level and clinical outcomes (Figure 4c). Cox proportional hazards model analysis revealed that tumor-infiltrating immune cells, excluding DC cells (log-rank *p* < 0.05), did not have a significant impact on clinical outcomes in LUAD. Our analysis indicates that, although the expression of BIRC5 slightly affects the infiltration of specific immune cells, the overall influence of BIRC5 on immune infiltration in LUAD tumors is not significant. Therefore, it is unlikely that immune infiltration alone can explain the poorer prognoses observed in LUAD patients with higher BIRC5 levels. Further confirmation of this speculation requires additional detailed experimental or clinical studies, as the analysis is solely based on a single database.

### 2.7. Relationship between DNA Methylation and Expression of BIRC5

**Figure 5:**
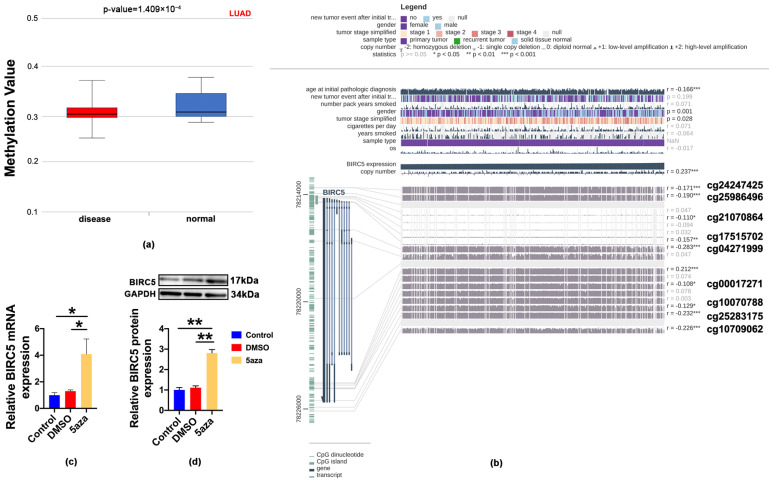
DNA methylation levels in BIRC5 were reduced in LUAD. (**a**) Methylation validation of BIRC5 using DiseaseMeth version 2.0. (**b**) MEXPRESS was used to visualize the association between gene expression and the methylation sites of BIRC5 DNA sequence. (**c**,**d**) mRNA and protein level of BIRC5 in the control, Dimethyl sulfoxide (DMSO) and 5-Azacytidine (5aza) treated A549 cells, values of * *p* < 0.05 and ** *p* < 0.01 vs. DMSO (n ≥ 4), respectively.

The analysis of DiseaseMeth version 2.0 showed that there was a notable decrease in the methylation of BIRC5 in LUAD tissues as opposed to paracancerous normal tissues (Figure 5a). Furthermore, we identified nine specific methylation sites (cg24247425, cg25986496, cg21070864, cg17515702, cg04271999, cg00017271, cg10070788, cg25283175 and cg10709062) in the DNA sequences of BIRC5 that exhibited a negative correlation with their expression levels using MEPRESS (Figure 5b) [19]. Then, we hypothesized that the high expression of the BIRC5 at mRNA and the protein level in LUAD were caused by abnormal modification of its DNA methylation. In order to verify this hypothesis, we further explored whether the expression of BIRC5 was enhanced with 5-Azacytidine (5aza), a specific methyltransferase inhibitor. The results showed that 5aza significantly upregulated BIRC5 expression at protein and mRNA level in A549 cells, suggesting that high expression of BIRC5 was induced with DNA demethylation (Figure 5c,d).

### 2.8. BIRC5 Silencing Significantly Suppressed the Tumor Growth In Vivo and In Vitro

**Figure 6:**
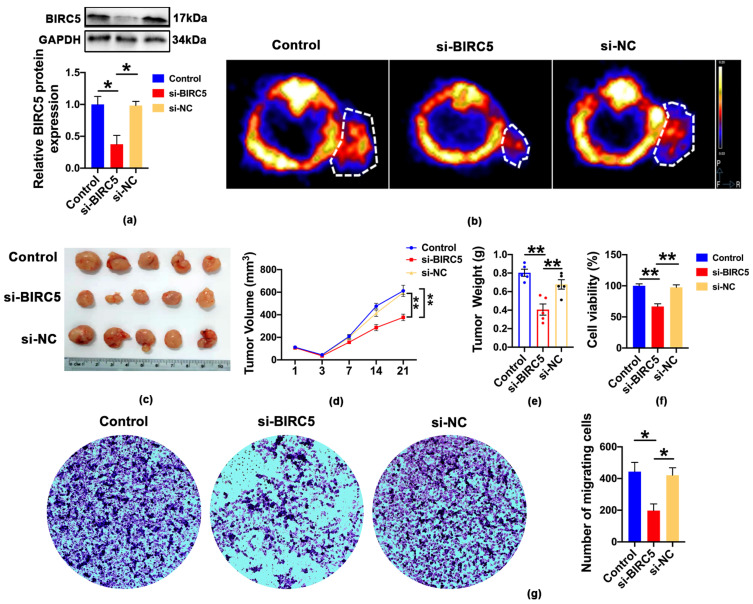
The BIRC5 deletion inhibits LUAD progression. (**a**) Western blot analysis of BIRC5 after transfection of BIRC5 siRNA into A549 cell, value of * *p* < 0.05 vs. si-NC (n = 3). (**b**) Representative micro-PET/CT images of tumor region after intravenous injection of ^18^F-FDG (n = 3). (**c**) Images of A549 xenografts tumor (n = 5). (**d**) Tumor volume of A549 xenografts tumor in each group, value of ** *p* < 0.01 vs. si-NC (n = 5). (**e**) Body weight of A549 xenografts tumor in each group, value of ** *p* < 0.01 vs. si-NC (n = 5). (**f**) Cell viability of BIRC5 inhibition measured with CCK-8 assay, value of ** *p* < 0.01 vs. si-NC (n = 3). (**g**) The cell migration was measured using transwell assay, value of * *p* < 0.05 vs. si-NC (n = 3).

To further clarify the function of BIRC5 in vivo, we generated BIRC5 knockdown cells using lentiviral transfection, the expression of BIRC5 was significantly decreased in the A549 cell line transfected with si-BIRC5, as confirmed with Western blot analysis (Figure 6a). Then, we divided the A549 cell lines into the following three groups: control, si-BIRC5 and si-NC. Consequently, we assessed the in vivo tumorigenetic ability of different groups of cells in xenografted mice. To further investigate this, we performed in vivo microPET imaging using ^18^F-FDG in tumor-bearing mice. Static PET images were acquired at 1 h after the injection of ^18^F-FDG for the control, si-BIRC5 and si-NC groups. In our study, we observed a significant reduction in tumor size in the BIRC5 deletion of A549-cell-xenografted mice compared to the NC group (Figure 6b). Additionally, we found that the tumor volume and weight were significantly decreased in the BIRC5’s deletion of A549-cell-xenografted mice compared to the NC group. (Figure 6c–e). CCK8 and transwell assays were conducted to assess the cell viability and migration capacity of A549 cells. The findings demonstrated that the inhibition of BIRC5 significantly suppressed the survival and migration of cells (Figure 6f,g). These data demonstrated that BIRC5 silencing displayed anti-cancer effects both in vivo and in vitro.

### 2.9. Caspase3 Was a Downstream Target of BIRC5 

**Figure 7:**
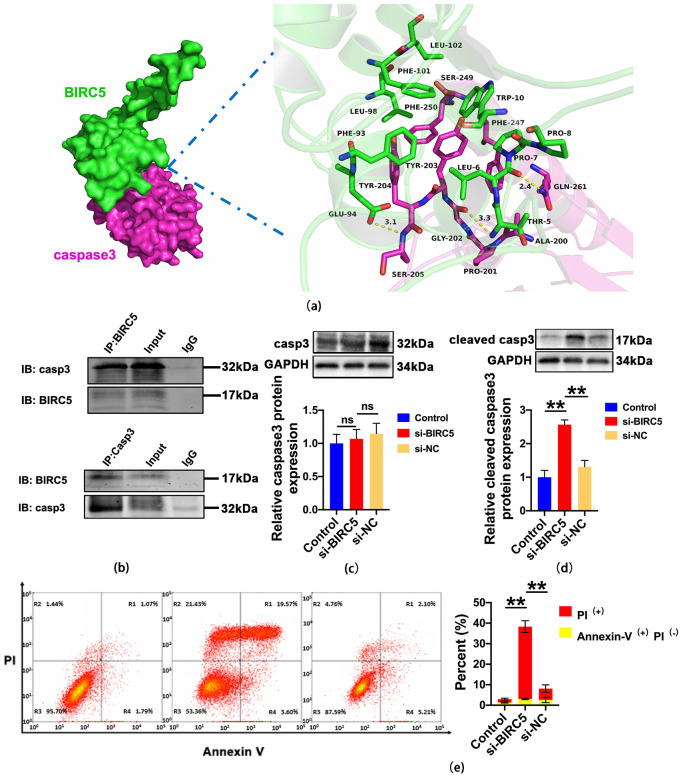
BIRC5 bond with Caspase3. (**a**) Docking analysis of the interaction between BIRC5 (green) and caspase3 (rose red). (**b**) Co-immunoprecipitation (Co-IP) validation of interaction between BIRC5 and caspase3 (n = 3). (**c**) The relative protein levels of caspase3 (n = 4). (**d**) The relative expression levels of cleaved caspase3 protein n = 3, value of ** *p* < 0.01 vs. si-NC, (n = 4). (**e**) The percentage of PI^+^ cells and Annexin V^−^FITC^+^ and PI^−^ were analyzed with flow cytometry, value of ** *p* < 0.01 vs. si-NC (n = 3).

To investigate the possible mechanism of BIRC5 in regulating LUAD, KEGG pathway enrichment analysis of BIRC5 was performed. The result shows that the enrichment term related to BIRC5 is mainly associated with cell apoptosis and platinum drug resistance (Figure S4a). Meanwhile, previous research studies conducted by others have shown that the BIRC5 is a member of the IAP (inhibitor of apoptosis) family, which can regulate caspase activation [20]. Then, we utilized molecular docking to investigate the binding mode between the human BIRC5 and apoptotic effector caspase3 using the ZDOCK server. The interaction between the human BIRC5 (green) and the caspase3 (rose red) was shown in Figure 7a. Detailed analysis showed that three hydrogen bond interactions occurred between the residue Glu-94 of the BIRC5 and the residue Ser-205 of the caspase3 (bond length: 3.1 Å); the residue Gly-202 of the BIRC5 and the residue Thr-5 of the caspase3 (bond length: 3.3 Å); and the residue Leu-6 of the BIRC5 and the residue Gln-261 of the caspase3 (bond length: 2.4 Å), which was the main binding affinity between the BIRC5 and the caspase3. Co-IP assays also showed that BIRC5 specifically interacted with caspase3 in A549 cells (Figure 7b). Moreover, we found that protein expression of cleaved caspase3 was increased after silencing BIRC5, but no change was found at the protein expression of caspase3 (Figure 7c,d). Next, we analyzed the regulation of BIRC5 on apoptosis of A549 cells with Annexin V-FITC/PI staining assay (Figure 7e). Further, statistical analysis of flow cytometry found that the percentage of Annexin V^+^/PI^−^ cells displayed no significant change in each group. Interestingly, the PI^+^ cells or the Annexin V^+^/PI^+^ cells, which were a phenotype of pyroptosis, were remarkably increased in BIRC5 deletion. Together with these results, we hypothesized that the inhibition of BIRC5 may have induced pyroptosis via cleavage of caspase3 in A549 cells.

### 2.10. GSDME Switch caspase3-Mediated Apoptosis to Pyroptosis Induced with BIRC5 Deletion

**Figure 8:**
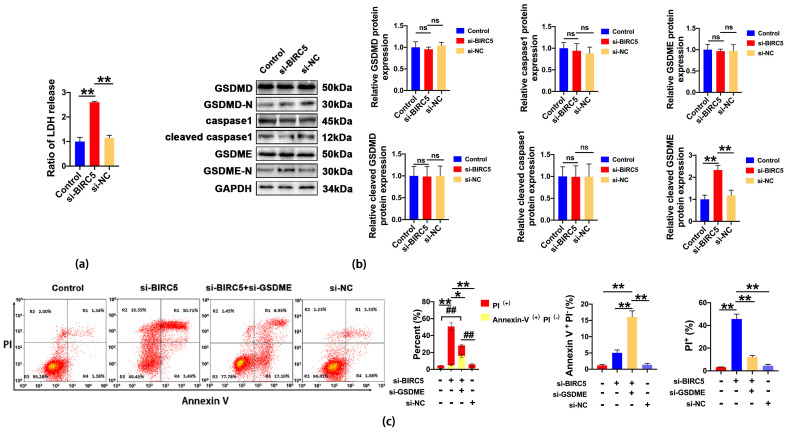
BIRC5 mediated apoptosis to pyroptosis. (**a**) The LDH content in the culture supernatants of A549 cells transfected with BIRC5 siRNA, value of ** *p* < 0.01 vs. si-NC (n = 5). (**b**) The relative expression levels of GSDMD, GSDMD-N, caspase1, cleaved caspase1, GSDME and GSDME-N in A549 cells transfected with BIRC5 siRNA using Western blot, value of ** *p* < 0.01 vs. si-NC (n ≥ 3). (**c**) The percentage of PI^+^ cells and Annexin V-FITC^+^ and PI^−^ in each group were analyzed with flow cytometry, values of * *p* < 0.05, ** *p* < 0.01, ## *p* < 0.01 (n = 3), respectively. ns, not significant.

In order to confirm our hypothesis, we tested indicators related to pyroptosis. The levels of LDH in the cell culture supernatants of A549 was significantly increased in the si-BIRC5 group (Figure 8a). Next, we analyzed the key signaling proteins in the canonical pyroptotic pathway including caspase1 and GSDMD. The results showed that the protein levels of GSDMD, GSDMD-N, caspase1 and cleaved caspase1 were not changed in the si-BIRC5 group. By contrast, another member of the gasdermin family, GSDME, was remarkably activated when BIRC5 was inhibited (Figure 8b). To further investigate whether GSDME was the downstream of caspase3 and if it involved in pyroptosis, we performed Annexin V-FITC/PI staining assay. The results revealed that the knockdown of GSDME markedly reduced the PI^+^ cells or the Annexin V^+^/PI^+^ cells and increased the percentage of Annexin V^+^/PI^−^ cells (Figure 8c). These results indicated that GSDME was a determinant in pyroptosis induced with BIRC5 inhibition in A549 cells.

## 3. Discussion

Lung cancers are the main cause of cancer incidence and malignant tumor-related mortality worldwide, in which a 5-year survival rate is very low (approximately 19%). LUAD is a kind of non-small cell lung cancer, accounting for approximately 40% of lung malignancies. Numerous studies over recent decades have identified a number of oncologic dependencies with the most frequent somatic mutations in LUAD being TP53, KRAS, EGFR, ALK, MET, STK11 and KEAP1. Recently, targeted therapies for cancer have made remarkable progress. Nonetheless, a substantial proportion of LUAD patients still lack targeted treatment options, either because there is a lack of known or currently targetable mutations or the mortality of LUAD remains largely unchanged. To address this issue, proteogenomic profiling is needed to screen out more candidate therapeutic targets and provide deeper mechanical insights into LUAD [21,22].

Bioinformatic analysis of transcription profiles using microarrays is a novel approach to explore pathogenesis of LUAD, identify disease biomarkers and discover therapeutic targets. Unlike simple visualization of gene expression analysis, a more comprehensive deep-scale analysis of LUAD based on genomic and post-translational modifications (PTM) can allow us to find out more novel molecular target consequences of epigenetic and genomic alterations. In this study, we performed a comprehensive genomic and DNA methylation modifications analysis of paired (patient matched) LUAD tumors and normal adjacent tissues. We downloaded the gene expression microarray dataset (GSE118370) and DNA methylation profiling microarray dataset (GSE139032) from the GEO database and conducted a bioinformatics study based on these two datasets. We obtained a total of 1377 MeDEGs associated with the pathogenesis of LUAD. Among them, a total of 510 upregulated and hypormethylated genes and 867 downregulated and hypermethylated genes were identified in lung adenocarcinoma tissues compared to adjacent normal lung tissues. The GO process and KEGG enrichment analysis were performed to investigate the biological function and signaling pathway of MeDEGs. We carried out hub genes by studying the functional correlations of MeDEG-encoded proteins with the help of the STRING database. Then, to validate the hub genes in MeDEGs, we further testified the expression and assessed the influence of candidate key genes on patient survival using clinical information in the TCGA dataset. After analyzing the DNA methylation of hub genes using DiseaseMeth (Table S1), we screened out eight novel hub genes including CCL20, MUC5B, ALDH3B2, TFF1, FA2H, BIRC5, ADRB2 and SLIT3 which were consistent with the sequencing data (GSE118370, GSE139032). Next, we verified the functionality of these hub genes through laboratory experiments. The results showed that BIRC5 is a new target for the diagnosis and treatment of lung adenocarcinoma, and that its expression was significantly elevated in LUAD and more positively associated with tumor progression [9,14,23,24,25].

The BIRC5, which is localized in the nucleus, is known for its multifunctional role in cell differentiation, proliferation, migration, and invasion of various tumor cell types. It plays a crucial role in facilitating tumor progression [26,27]. BIRC5, a suppressor of apoptosis encoding the protein Survivin, is a mitotic spindle checkpoint gene located on chromosome 17 that is overexpressed in most cancer cells and its high expression is associated with worse survival [28]. Moreover, research studies have shown that BIRC5 is widely used in the treatment of multiple cancers as a biomarker of chemotherapy resistance, which increases metastatic activity and tumor recurrence risk [29]. Numerous studies have shown that the elevation of BIRC5 induced multi-drug resistance in various cancers. Conversely, silencing BIRC5 could inhibit the proliferation of tumor cells and promote apoptosis [26,30]. Recently, researchers have explored the combination of BIRC5 inhibitors with immune checkpoint inhibitors or monoclonal antibodies, which has shown promising results in inhibiting the growth of mouse xenograft tumors [31]. It is generally believed in previous studies that the BIRC5 gene is a driving force for tumorigenesis and a known inhibitor of apoptosis [28]. However, in our study, the flow cytometry results showed that the percentage of Annexin V-FITC and PI double-positive cells and PI single-positive cells was remarkably elevated after the BIRC5 inhibition compared with the control group in A549 cells. Therefore, these data suggested that the BIRC5 inhibition may have induced pyroptosis in A549 cells. Pyroptosis, also known as cell inflammatory necrosis, is a kind of programmed cell death that is characterized by cell size increase and formation of pores and plasma membrane disruption, which affects membrane integrity and increases the release of cell contents [32]. This hypothesis was confirmed through an LDH release assay, which showed a significant increase in LDH release in the BIRC5 inhibition group. Mechanically, a key feature of pyroptosis is the involvement of gasdermin proteins as the main executive protein in cell death [33]. The classic activation pathway of pyroptosis is triggered with the activation of caspase1, leading to the cleavage of the GSDMD [34]. However, Western blot results revealed that the BIRC5 inhibition did not significantly affect the expression of cleaved caspase1 and GSDMD-N. The majority of BIRC5 was found to inhibit cell death by blocking the activation of caspase3, which aligns with our findings. Additionally, Shao et al. discovered that chemotherapy drugs can trigger pyroptosis through caspase3-mediated cleavage of Gasdermin E [35]. Our results corroborate with this, as we observed a significant increase in the N-terminal fragments of Gasdermin E when BIRC5 was deleted. Furthermore, when GSDME was knocked down, it hindered the pyroptosis induced with BIRC5 inhibition.

In summary, our findings revealed the BIRC5 was identified and confirmed as a potential prognostic biomarker for LUAD. We found that inhibiting BIRC5 led to the induction of pyroptosis in lung adenocarcinoma cells through the caspase3/GSDME pathway. This study challenges the conventional understanding of programmed cell death, specifically, the role of caspase3 as the hallmark of apoptosis. Based on our findings, we hypothesize that the expression or expression level of GSDME plays a crucial role in determining the form of cell death in caspase3-activated cells. Pyroptosis was quickly induced after the cleavage of caspase3 in GSDME-enriched cells; however, apoptosis was dominant in low GSDME expression cells. While our study is perspective and efficient in identifying hub genes in lung adenocarcinoma, it is important to acknowledge the presence of certain limitations. For instance, it would be advantageous to incorporate a broader range of lung adenocarcinoma cell lines to explore the correlation between BIRC5 inhibition-induced pyroptosis and cellular GSDME expression levels. This would help to determine if the inhibition of BIRC5 induces pyroptosis via the caspase3-GSDME pathway in all lung adenocarcinoma cell lines. Furthermore, the increasing number of genetic screening results for clinical lung adenocarcinoma patients reveals a significant variability among individuals. However, it is important to note that our study evaluated only five clinical samples, which may limit the comprehensiveness of our assessment. In future research, we recommend increasing the sample size and include different types of samples. This will enable us to gain a more comprehensive understanding of the role of BIRC5 in lung adenocarcinoma and identify new personalized treatment targets for the clinical diagnosis and management of lung cancer. Finally, it is crucial to explore the influence of DNA methylation on BIRC5/Survivin expression. In future experiments, we plan to utilize sequencing and other techniques to identify key regulatory enzymes responsible for mediating methylation changes in BIRC5 [36]. This study conducted a comprehensive bioinformatics analysis of MeDEGs and identified BIRC5 as a potential prognostic factor for LUAD. The findings of this study suggest that the inhibition of BIRC5 can induce cell death through caspase3/GSDME-mediated pyroptosis in lung adenocarcinoma. These findings offer potential therapeutic targets and candidate biomarkers and contribute to our understanding of the pathogenesis of lung adenocarcinoma.

## 4. Materials and Methods

### 4.1. RNA-Seq and Microarray Data

In the initiation of this study, we downloaded the lung adenocarcinoma methylation microarray and expression microarray datasets from publicly available Gene Expression Omnibus database (GEO) of NCBI (https://www.ncbi.nlm.nih.gov/gds/; accessed on 9 January 2021). Finally, expression microarray datasets (GSE118370) and methylation microarray datasets (GSE139032) were used for biological information analysis. A total of 6 pairs of LUAD tumors and matched non-tumor tissues were enrolled in GSE118370 (Platform: GPL570, Affymetrix Human Genome U133 Plus 2.0 Array). The DNA methylation dataset (GSE139032), which was based on the GPL8490 platform (Illumina HumanMethylation27 BeadChip), included 77 primary lung adenocarcinoma cancer tissues and 77 adjacent normal lung tissues.

### 4.2. Identification of MeDEGs

The DEGs between tumor and non-cancerous samples in expression microarray datasets and DMGs in methylation microarray datasets were screened using GEO2R, which is an online analyzing tool that allows users to compare two groups of samples under the same experimental conditions in a GEO Series. Adj. *p*-value < 0.01 and |*t*| > 2 were set as the threshold values to screen aberrant expression genes. Overlapping genes from GSE1118370 and GSE139032 were identified with FunRich software (Functional Enrichment analysis tool). As a result, the overlapping genes were filtered out as methylation-regulated differentially expressed genes (MeDEGs), including upregulated and hypomethylated, and downregulated and hypermethylated genes.

### 4.3. Functional and Pathway Enrichment Analysis of MeDEGs

FunRich (Functional Enrichment analysis tool) is an online analysis tool consisting of an integrated biological knowledgebase and analytic tools providing a comprehensive set of functional annotation information from large gene/protein lists. In order to explain the function of the two gene lists, the Gene Ontology (GO) function and the Kyoto Encyclopedia of Genes and Genomes (KEGG) pathways were determined using FunRich, and a *p* value of <0.05 was considered statistically significant.

### 4.4. Construction of Protein–Protein Interaction (PPI) Network, Module Analysis and Hub Genes Screening

The PPI network of previously identified methylation-regulated differentially expressed genes was predicted and built using STRING online database (http://string-db.org; accessed on 10 January 2021). An interaction with a combined score of >0.4 and *p* < 0.05 were seen to be considered as the threshold value. Cytoscape software (version 3.7.0; http://www.cytoscape.org/; accessed on 10 February 2021) is an open-source bioinformatics software platform that is utilized to integrate, analyze and visualize molecular interaction networks. The CytoHubba of Cytoscape is an application for screening the hub genes based on the total amount of degrees it possesses. A degree of more than 10 was used as cutoff criteria for hub gene identification.

### 4.5. Validation and Survival Analysis of Hub Genes in Other Datasets

Gene Expression Profiling Interactive Analysis (GEPIA; http://gepia.cancer-pku.cn/; accessed on 16 February 2021) is a straightforward and easy-to-use online tool for the integration and visualization of gene expression based on The Cancer Genome Atlas (TCGA) and the Genotype-Tissue Expression (GTEx). This database was used to perform the expression level, tumor staging and overall survival analysis of hub genes in cancer. DNA methylation was validated in DiseaseMeth (http://biobigdata.hrbmu.edu.cn/diseasemeth/; accessed on 19 February 2021), a database of DNA Methylation in human cancer based on The Cancer Genome Atlas (TCGA) database. 

### 4.6. Cell Culture and Cell Transfection

Human A549 (lung carcinoma) cells were purchased from the American Type Culture Collection (ATCC) and maintained in RPMI1640 (Hyclone, Logan, UT, USA) medium containing 10% fetal bovine serum (Gibco, Waltham, MA, USA) and 1% penicillin-streptomycin-glutamine (Solarbio, Beijing, China). The cells were incubated at 37 °C in humidified incubator containing 5% CO_2_. Cell transfection was performed after the cells reached 60% of confluence. ShRNA knockdown for GSDME and negative controls were purchased from GENECHEM (Shanghai, China). Transfection of plasmids was carried out by mixing them with Lipofectamine^TM^ 2000 reagent (Invitrogen, Carlsbad, CA, USA). ShRNA knockdown lentivirus for BIRC5 and their controls were synthesized with GENECHEM (Shanghai, China). The A549 cell line was infected with lentiviral particles to knockdown BIRC5. For the negative control, only a lentiviral vector was used. A549 cells were cultured for 48 h following infection and selected via fluorescence using flow cytometry (BECKMAN COULTER MoFlo XDP, Brea, CA, USA) for subsequent experiments.

### 4.7. Cell Viability Assay

A549 cells were transfected with lentiviral particles to knockdown BIRC5. After 48 h, the cells were collected and seeded at a density of 5 × 10^3^ cells per well into 96-well plates in DMEM containing 10% FBS and grown overnight. On the second day, 10 μL CCK-8 (APExBIO, Houston,TX, USA) solution was added to each well and incubated at 37 ℃ with 5% CO_2_ for 2 h. The absorbance values were measured at 450 nm wavelength using an enzyme-linked immunosorbent assay reader (BioTek, Thermo Fisher Scientific, Waltham, MA, USA). Results are presented as percentages relative to control cells. 

### 4.8. LDH Release Assay

A549 cell culture medium was collected from various treatment groups and centrifuge at 1000 rpm for 5 minutes to eliminate impurities and cellular debris. The levels of released LDH in the A549 cell culture supernatants were measured with the LDH cytotoxicity assay kit (Solarbio, Beijing) according to the manufacturer’s instructions. The absorbance was measured at 490 nm with a microplate reader (BioTek, Thermo Fisher Scientific, Massachusetts, USA).

### 4.9. Western Blot Analysis

The tumor tissue and cells were lysed with RIPA buffer (Solarbio, Beijing, China) and contained a protease inhibitor. Total protein concentrations were quantified with BCA protein assay kit (Solarbio, Beijing, China). Equal amounts of protein samples were loaded in SDS-PAGE and transferred onto polyvinylidene fluoride membranes (PVDF, Millipore, Burlington, MA, USA). Then, the membrane was blocked with quick blocking solution (Epizyme, Shanghai, China) at room temperature for 15 min and incubated at 4 ℃ overnight with anti-BIRC5 rabbit monoclonal antibody (1:500; #2808S, Cell Signaling Technology, Danvers, MA, USA); anti-caspase1 rabbit monoclonal antibody (1:1000; ab207802, abcam, Cambridge, UK); anti-caspase3 rabbit monoclonal antibody (1:1000; ab32351, abcam, Cambridge, UK); anti-GSDMD rabbit monoclonal antibody (1:1000; ab209845, abcam, Cambridge, UK); anti-GSDM rabbit monoclonal antibody (1:1000; ab222408, abcam, Cambridge, UK) and anti-GAPDH rabbit monoclonal antibody (1:1000; TA309157, ZSGB-BIO, Beijing, China). Next, the membrane was incubated with HRP-conjugated secondary antibody for 2 h at room temperature (1:10,000; Epizyme, Shanghai, China). Subsequently, the membrane was incubated with an HRP-conjugated secondary antibody for 2 h at room temperature. The visualization of the membrane was achieved by using ECL detection reagent (BMU102-CN, abbkine, Wuhan, China) and the image was captured using the Bio-Rad ChemiDoc XRS+ chemiluminescence imaging system (Bio-Rad, Hercules, CA, USA). 

### 4.10. RNA Extraction and qRT-PCR

Total RNA was extracted from tissues using the Trizol reagent (Invitrogen, Carlsbad, CA, USA) according to manufacturer’s protocol, and 1 µg RNA was reversely transcribed into cDNA with PrimeScript™ RT reagent kit (RR047A, Takara, Otsu City, Shiga Prefecture, Japan) according to the manufacturer’s instructions. Quantitative RT-PCR was performed with SYBR Green (4913914001, Roche, Basel, Switzerland) using QuantStudio3 Real-Time PCR instrument (Thermo Fisher Scientific, Massachusetts, USA). PrimerDesign was performed with Invitrogen (Invitrogen, Carlsbad, CA, USA) and GAPDH was used as house-keeping gene. Fold changes were calculated using the 2^−ΔΔCT^ method. A full list of the primers used in qPCR analyses is shown in Table S2.

### 4.11. Flow Cytometry Analysis

The treatment of A549 cells was transfected for 48 h and apoptosis assays were conducted using a BD Pharmingen Annexin V/PI Apoptosis Detection Kit I (BD Biosciences, Franklin Lake, NJ, USA). Briefly, A549 cells with different treatments were harvested, washed with PBS and diluted to a concentration of 1 × 10^4^ in 100 μL 1 × binding buffer. A total of 5 μL FITC-conjugated annexin V were added to the cell suspension and incubated for 5 min in 5 μl propidium iodide (PI) for 15 min at 37 °C in darkness. The cells were analyzed with flow cytometry (BECKMAN COULTER MoFlo XDP, Brea, CA, USA).

### 4.12. Immunohistochemistry

Lung adenocarcinoma tumor samples and paracancer tissue samples were collected from patients with primary lung adenocarcinoma cancer from The Third Affiliated Hospital of Harbin Medical University. All samples were obtained with informed patients’ consent and approved by the Ethics Committee of Harbin Medical University Pharmacy College (No. IRB5004822). Formalin-fixed tissues were paraffin embedded and sectioned; then, the sections were deparaffinized in xylene, hydrated in graded alcohol solutions and heated (100 °C) for 15 min in antigen retrieval citra solution (pH 6.0) for antigen retrieval. A total of 3% hydrogen peroxide solution (Sigma-Aldrich, Saint Louis, MO, USA) in PBS with 0.3% Triton X-100 was used to block endogenous peroxidase activity for 10 min at room temperature, washed twice with PBS and blocked with goat serum for 15 minutes at 37 ℃. The sections were incubated with BIRC5 primary antibody (1:100; #2808S, Cell Signaling Technology, USA) overnight at 4 ℃, followed by 2 h incubation with the corresponding horseradish peroxidase-conjugated secondary antibody. Then, the images were obtained and analyzed with microscopy (X71, Olympus, Tokyo, Japan).

### 4.13. Animals and Animal Model 

Six-week-old BALB/c nude female mice were purchased from Beijing Vital River Laboratory Animal Technology Co., Ltd (Vital River, Beijing, China). Mice were maintained in sterilized cages with 12/12 h light/dark cycle environment, 22 ℃ constant temperature and humidity of 55–60%, and were fed autoclaved food and water ad libitum. All animal experimental procedures and protocols conformed to the guide rules of the Animal Ethics Committee at Harbin Medical University. For xenografts, total 24 BALB/c nude mice were randomly divided into three groups (n = 8). The 2 × 10^7^ of A549 cells (control, si-BIRC5, si-NC) in 200 uL cold PBS (Hyclone Laboratories, Logan, UT, USA) were subcutaneously injected into the right shoulder of mice to form a xenografts animal model. Tumor volumes were measured every day after the inoculation. Tumor volume was measured with a digital caliper and calculated with the following equation: V = L × W^2^ × 0.5 (V is the volume, L is the length, and W is the width). Tumor-bearing mice were sacrificed under anesthesia 21 days after tumor inoculation and tumor tissue was explanted for weight measurement. For the orthotopic mouse model, A549 cells were harvested and resuspended in PBS medium at a final concentration of 10^7^ cells/mL. The animals were anesthetized with an intramuscular administration of 25 μL Zoletil™ 50. A LS-BA m blade assembly mouse laryngoscope (Penn-Century, Inc., Wyndmoor, PA, USA) was then used to expose the glottis, and a homemade syringe with a diameter of 0.4 mm was used to reach into the main trachea. Subsequently, tumor cells (50 μL) were inoculated into the lungs. After orotracheal intubation, the animals were kept in a stable environment with controlled temperature and humidity, as described above. The investigation conformed to the guide for the rules of the Declaration of Helsinki of 1975, revised in 2013, and was approved by the Institutional Animal Care and Use Committee of Harbin Medical University (No. IRB5004822).

### 4.14. Micro-PET/CT Imaging

FDG was produced routinely in GE TracerLab with a commercially available module following the standard protocols with quality control and radiochemical purity reaching 99%. Six hours before the imaging session, the mice were fasted, and water was given ad libitum. Approximately 200 μCi ^18^F-FDG was injected into tumor-bearing mice via the tail vein. A total of 15 min static scanning was performed at 1 h after injection with mice under isoflurane (R510-22-10, RWD, Shenzhen, China) anesthesia using a SuperArgus small-animal PET/CT scanner (Sedecal, Madrid, Spain). Images were reconstructed and regions of interest (ROI) were manually drawn around tumors.

### 4.15. Cell Migration Assays

For the migration studies, A549 cells were transfected with lentiviral particles to knockdown BIRC5. After 48 h, 1 × 10^5^ A549 cells were resuspended in 100 µL serum-free 1640 RPMI medium and transferred into the upper chamber of 24-well transwell chamber (Corning, New York, NY, USA). Then, 500 μL complete 1640 RPMI with 10% FBS as a chemoattractant was added to the lower chambers. The plates were incubated at 37 °C with 5% CO_2_ for 24 h. Next, the cells adhering to the upper surface of the membrane were removed with a cotton swab. The migration cells, which adhered to the lower surface, were stained with 0.5% crystal violet for 15 min according to the manufacturer’s instructions. The images were captured with microscopy (X71, Olympus, Tokyo, Japan). The number of cells was counted randomly at ×200 magnification.

### 4.16. Statistical Analyses 

Statistical analyses were performed using GraphPad Prism 8. Data are presented as mean ± SEM. Student’s *t*-test was performed to analyze data for experiments with two groups. One-way ANOVA was used to compare multiple experimental groups. A *p* value < 0.05 was considered as statistically significant.

## Data Availability

The data presented in this study are available from the corresponding author upon reasonable request.

## Supplementary Materials

The following supporting information can be downloaded at: https://www.mdpi.com/article/10.3390/ijms241914663/s1.
