# Supplementary material for: BIRC5 Inhibition Is Associated with Pyroptotic Cell Death via Caspase3-GSDME Pathway in Lung Adenocarcinoma Cells
Source: Int J Mol Sci. 2023 Sep 28;24(19):14663. doi: 10.3390/ijms241914663 (PMC10572361; doi:10.3390/ijms241914663)
Supplement: Supplementary file 1 [file ijms-24-14663-s001.zip › Figures and Tables legends of supplementary.pdf]

**Figure S1.** Go and KEGG enrichment analysis. (a) Biological process (BP) analysis of upregulated- hypomethylation genes. (b) Biological process of downregulated-hypermethylation genes. (c) Molecular function (MF) analysis of upregulated-hypomethylation genes. (d) Molecular function of downregulated-hypermethylation genes. (e) Cellular component (CC) analysis of upregulated-hypomethylation genes. (f) Cellular component of downregulated-hypermethylation genes. (g) The most significantly enriched KEGG pathway of upregulated-hypomethylation genes. (h) KEGG pathway of downregulated-hypermethylation genes.

**Figure S2.** The PPI network and the validation of gene expression and survival analysis for downregulated and hypermethylated genes. (a) The PPI network analysis of the 42 downregulated-hypermethylated genes, visualized by Cytoscape tool. (b) Expression validation of downregulated-hypermethylation genes. (c) Kaplan–Meier overall survival curves were for survival analysis of downregulated-hypermethylation genes.

**Figure S3.** Correlation between the expression of candidate genes and tumor stage in lung adenocarcinoma.

**Figure S4.** KEGG pathway enrichment analysis of BIRC5.

**Table S1.** DNA methylation validation of the key hub genes.

**Table S2.** Gene primer sequences.
