# Supplementary material for: BIRC5 Inhibition Is Associated with Pyroptotic Cell Death via Caspase3-GSDME Pathway in Lung Adenocarcinoma Cells
Source: Int J Mol Sci. 2023 Sep 28;24(19):14663. doi: 10.3390/ijms241914663 (PMC10572361; doi:10.3390/ijms241914663)
Supplement: Supplementary file 1 [file ijms-24-14663-s001.zip › Table S1.pdf]

| Disease Name | Genomic region | Transcript | Gene | *p*-value | Mean Methyl Disea - mean Methyl Normal |
|---|---|---|---|---|---|
| Lung adenocarcinoma[LUAD] | chr5:148204155-148206655 | NM_000024 | ADRB2 | 1.650e-06 | 0.012 |
| Lung adenocarcinoma[LUAD] | chr11:67441602-67444102 | NM_000695 | ALDH3B2 | 7.678e-07 | -0.072 |
| Lung adenocarcinoma[LUAD] | chr7:36427411-36429911 | NM_001284301 | ANLN | 3.156e-01 | -0.003 |
| Lung adenocarcinoma[LUAD] | chr17:76208276-76210776 | NM_001012270 | BIRC5 | 1.409e-04 | -0.012 |
| Lung adenocarcinoma[LUAD] | chr2:228676557-228679057 | NM_004591 | CCL20 | 1.413e-10 | -0.063 |
| Lung adenocarcinoma[LUAD] | chr5:59995493-59997993 | NM_018369 | DEPDC1B | 1.704e-01 | 0.003 |
| Lung adenocarcinoma[LUAD] | chr9:130608946-130611446 | NM_001278138 | ENG | NA | NA |
| Lung adenocarcinoma[LUAD] | chr16:74808229-74810729 | NM_024306 | FA2H | 1.393e-04 | -0.006 |
| Lung adenocarcinoma[LUAD] | chr11:1242294-1244794 | NM_002458 | MUC5B | 1.626e-12 | -0.041 |
| Lung adenocarcinoma[LUAD] | chr17:62406583-62409083 | NM_000442 | PECAM1 | NA | NA |
| Lung adenocarcinoma[LUAD] | chr5:168727633-168730133 | NM_001271946 | SLIT3 | 1.625e-12 | 0.119 |
| Lung adenocarcinoma[LUAD] | chr21:43786144-43788644 | NM_003225 | TFF1 | 9.129e-12 | -0.080 |
| Lung adenocarcinoma[LUAD] | chr17:76182785-76185285 | NM_003258 | TK1 | 0.000e+00 | -0.041 |
