# Supplementary material for: BIRC5 Inhibition Is Associated with Pyroptotic Cell Death via Caspase3-GSDME Pathway in Lung Adenocarcinoma Cells
Source: Int J Mol Sci. 2023 Sep 28;24(19):14663. doi: 10.3390/ijms241914663 (PMC10572361; doi:10.3390/ijms241914663)
Supplement: Supplementary file 1 [file ijms-24-14663-s001.zip › Table S2.pdf]

Gene	Sequence (5’-3’)
Hsa-BIRC5	Forward: AGGACCACCGCATCTCTACAT
	Reverse: AAGTCTGGCTCGTTCTCAGTG
Hsa-CCL20	Forward: TGCTGTACCAAGAGTTTGCTC
	Reverse: CGCACACAGACAACTTTTTCTTT
Hsa-MUC5B	Forward: GCCTACGAGGACTTCAACGTC
	Reverse: CCTTGATGACAACACGGGTGA
Hsa-ALDH3B2	Forward: ATGAAGGATGAACCACGGTCC
	Reverse: CCCCGTGAAAGACAGAATTGT
Hsa-TFF1	Forward: CGCCTCTACGACCTCTCCA
	Reverse: GGTGTCGTCGAAACAGCAG
Hsa-FA2H	Forward: CGCCTCTACGACCTCTCCA
	Reverse: TGGTTCCATAGCAGGATCTGTC
Hsa-ADRB2	Forward: TTGCTGGCACCCAATAGAAGC
	Reverse: CAGACGCTCGAACTTGGCA
Hsa-SLIT3	Forward: GTCAGCGTCATCGAGAGAGG
	Reverse: TTCGGCGTGCTCTGGAAAAG
Hsa-GAPDH	Forward: GGAGCGAGATCCCTCCAAAAT
	Reverse: GGCTGTTGTCATACTTCTCATGG
