# Supplementary figures and images for: BIRC5 Inhibition Is Associated with Pyroptotic Cell Death via Caspase3-GSDME Pathway in Lung Adenocarcinoma Cells
Source: Int J Mol Sci. 2023 Sep 28;24(19):14663. doi: 10.3390/ijms241914663 (PMC10572361; doi:10.3390/ijms241914663)

## Upregulated- hypomethylation

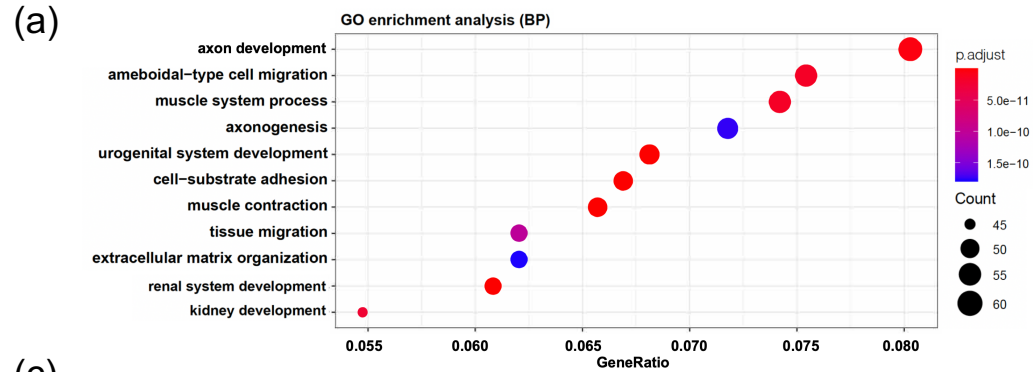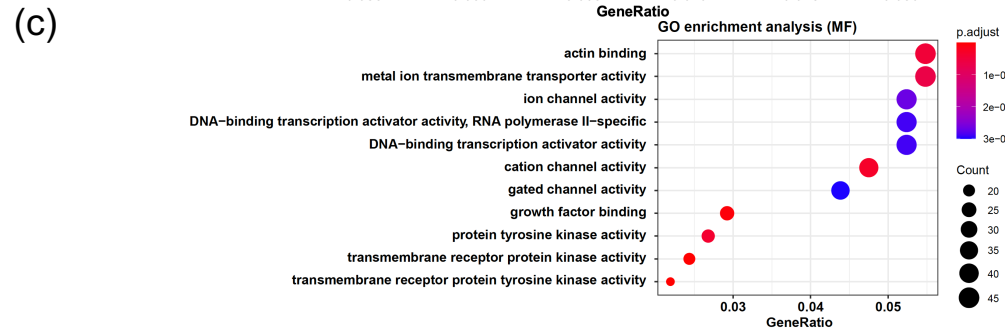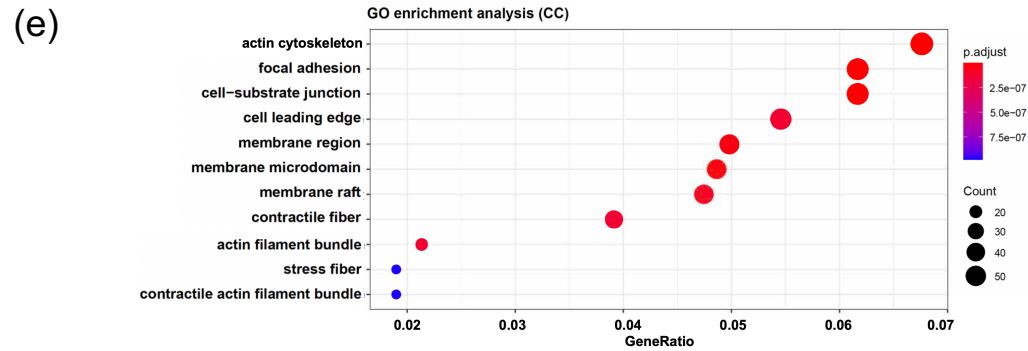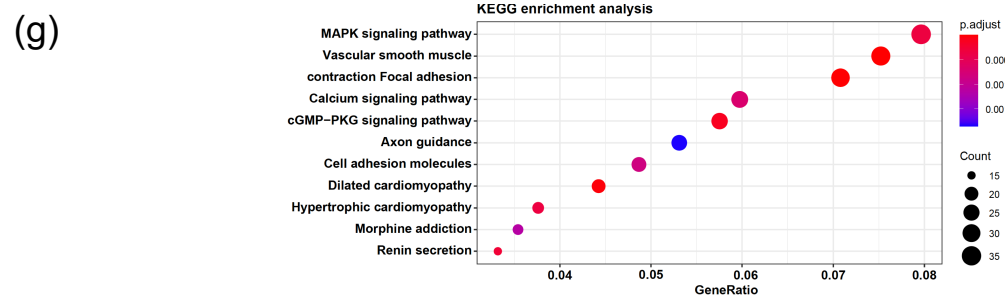

## Downregulated-hypermethylation

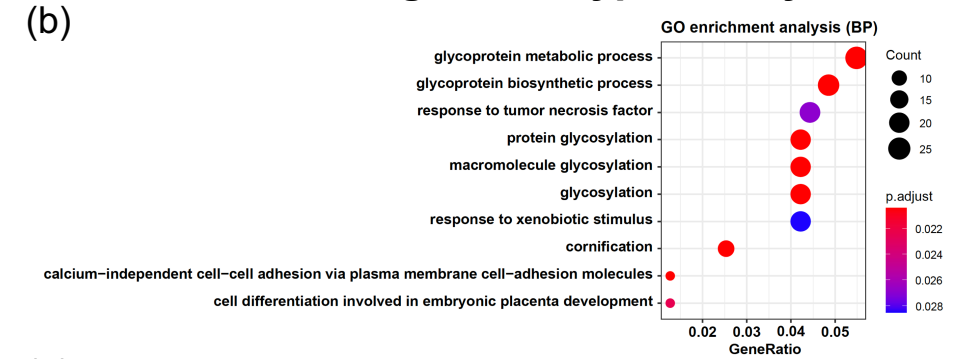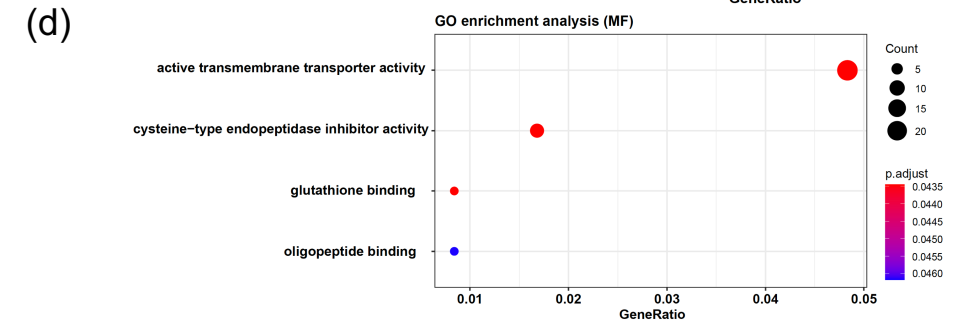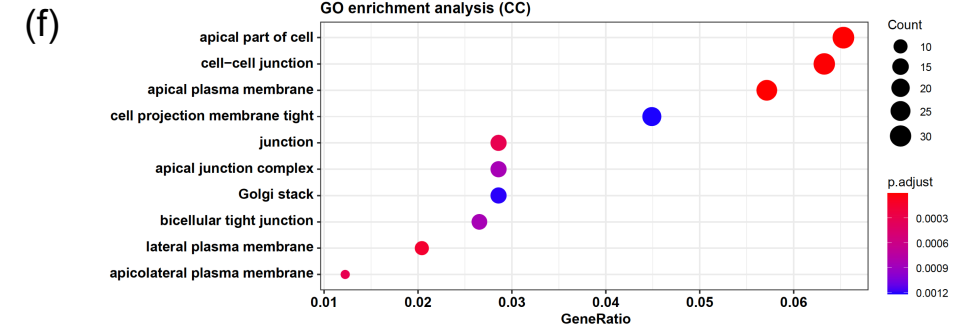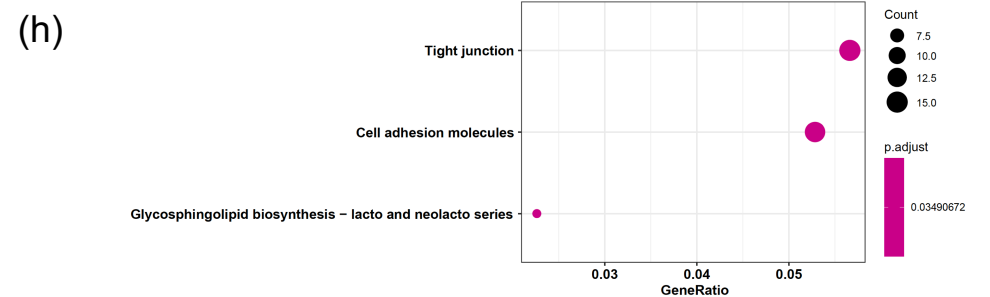

Supplement: Supplementary file 1 [file ijms-24-14663-s001.zip › Figure S1.pdf]

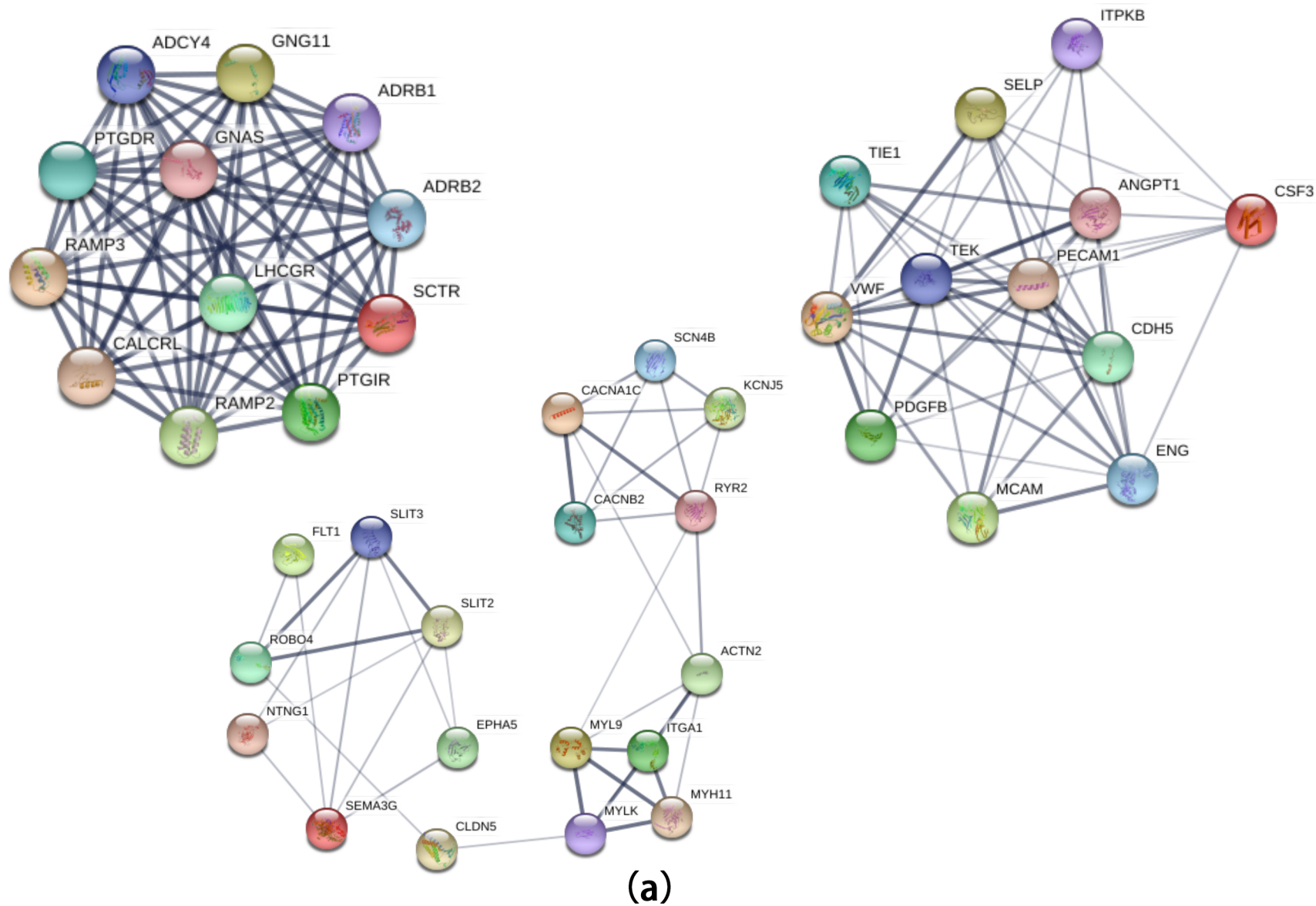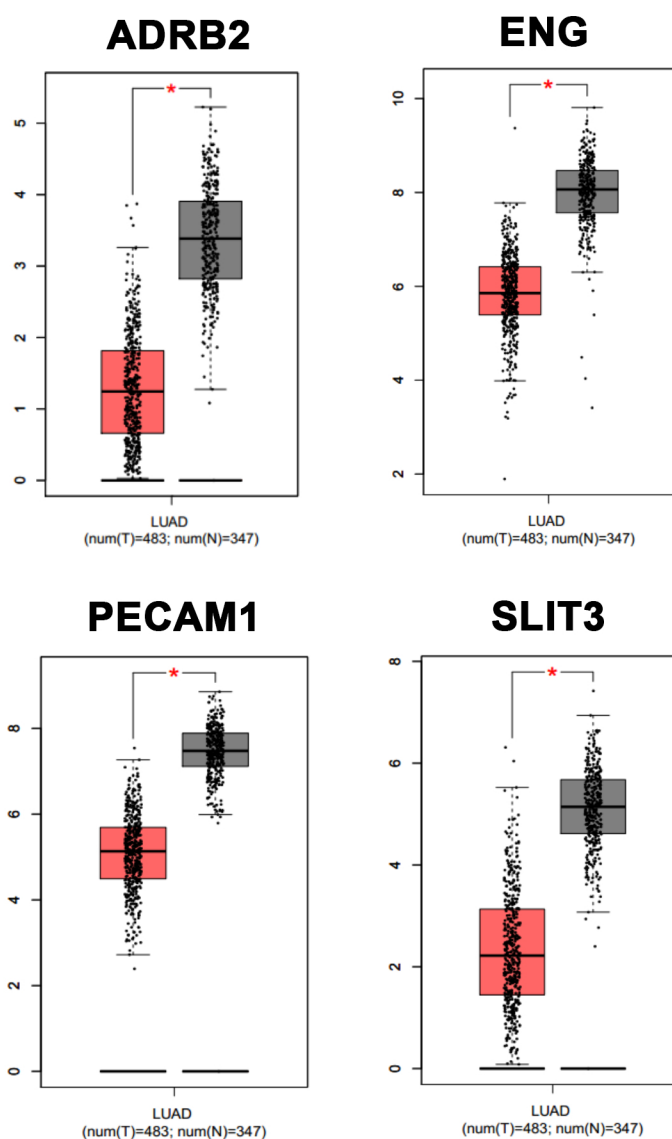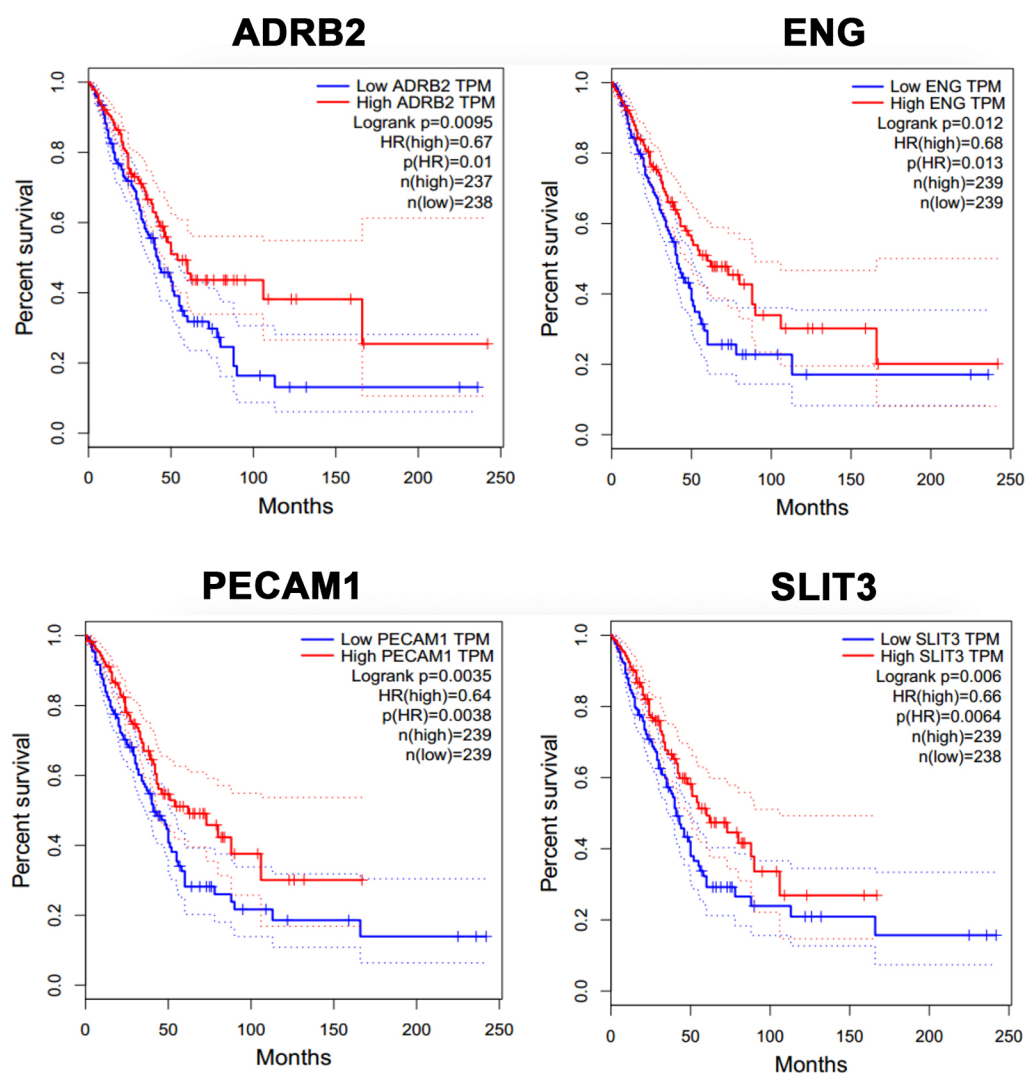

■ Tumor ■ Normal

Supplement: Supplementary file 1 [file ijms-24-14663-s001.zip › Figure S2.pdf]

**CCL20**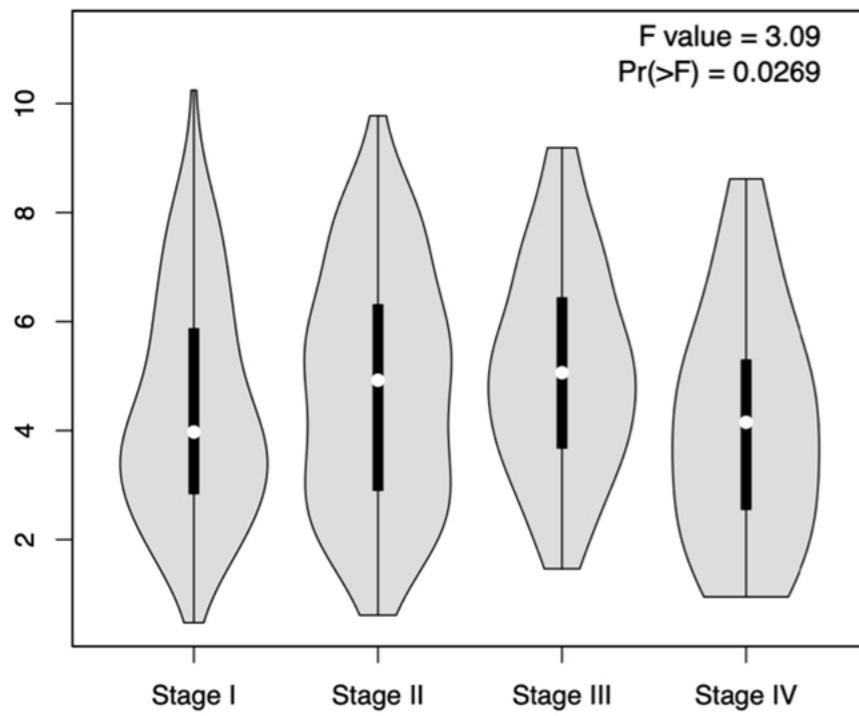**(a)****MUC5B**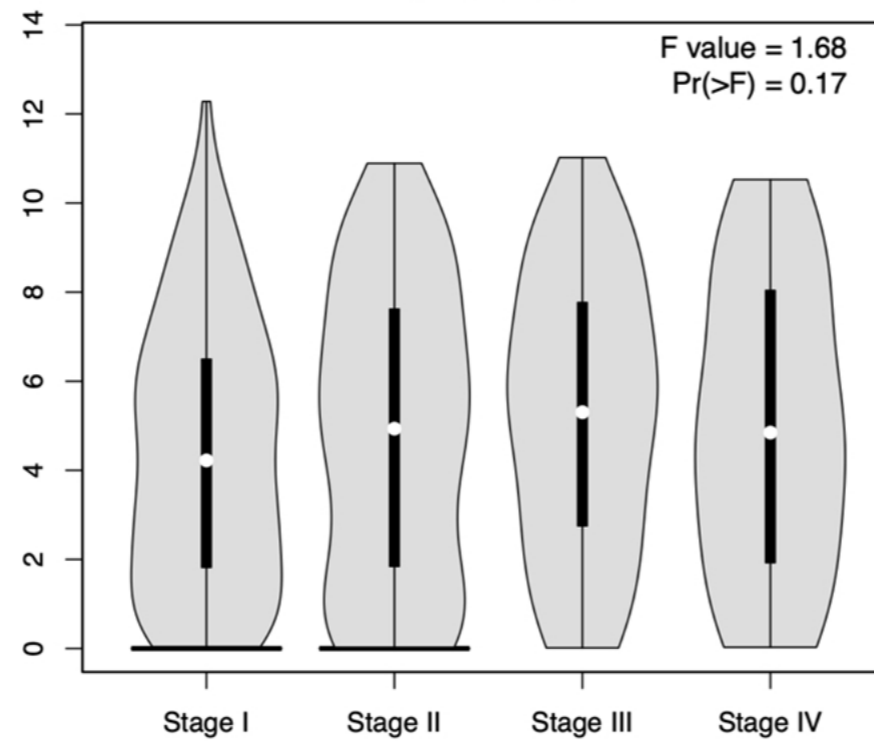**(b)****BIRC5**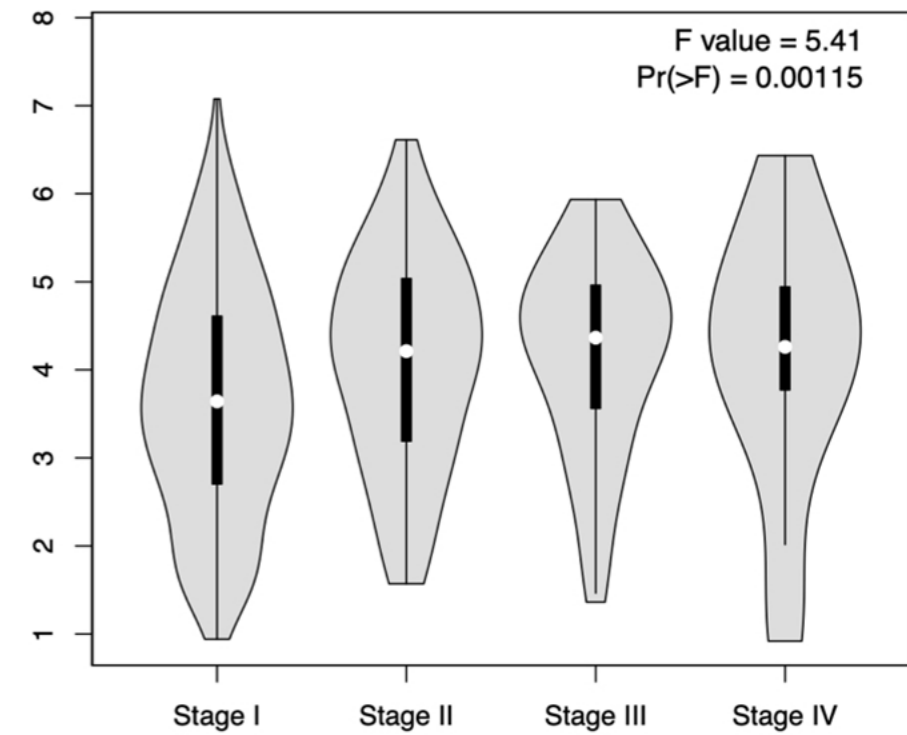**(c)****ADRB2**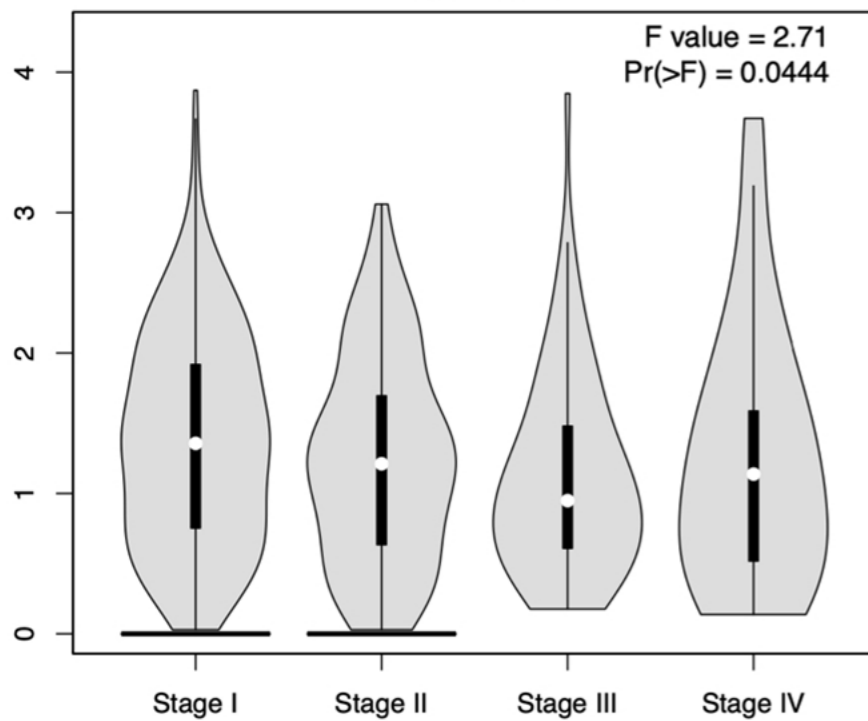**(d)****SLIT3**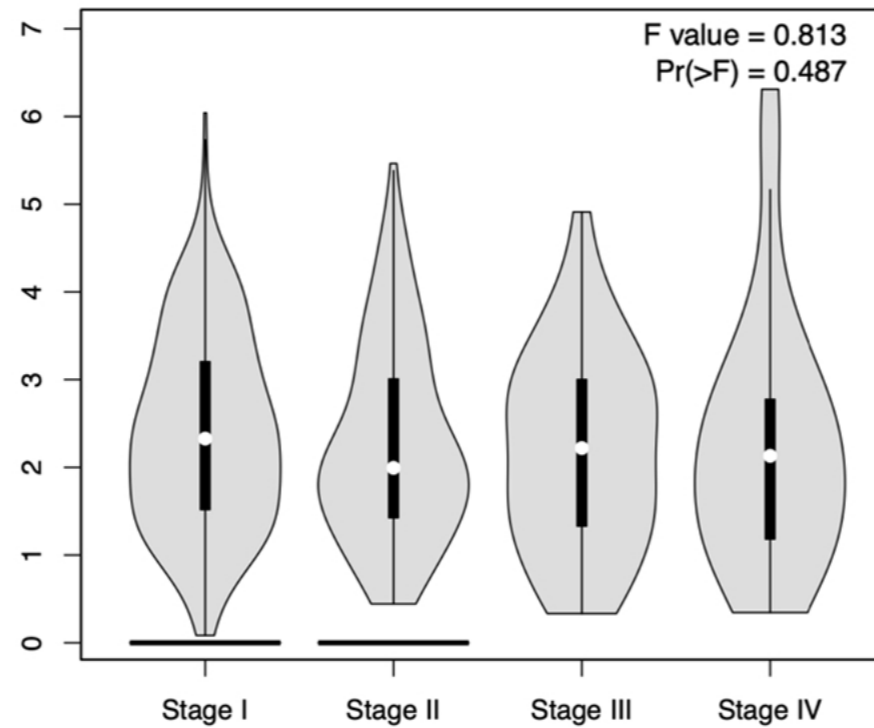**(e)**

Supplement: Supplementary file 1 [file ijms-24-14663-s001.zip › Figure S3.pdf]

# KEGG enrichment analysis

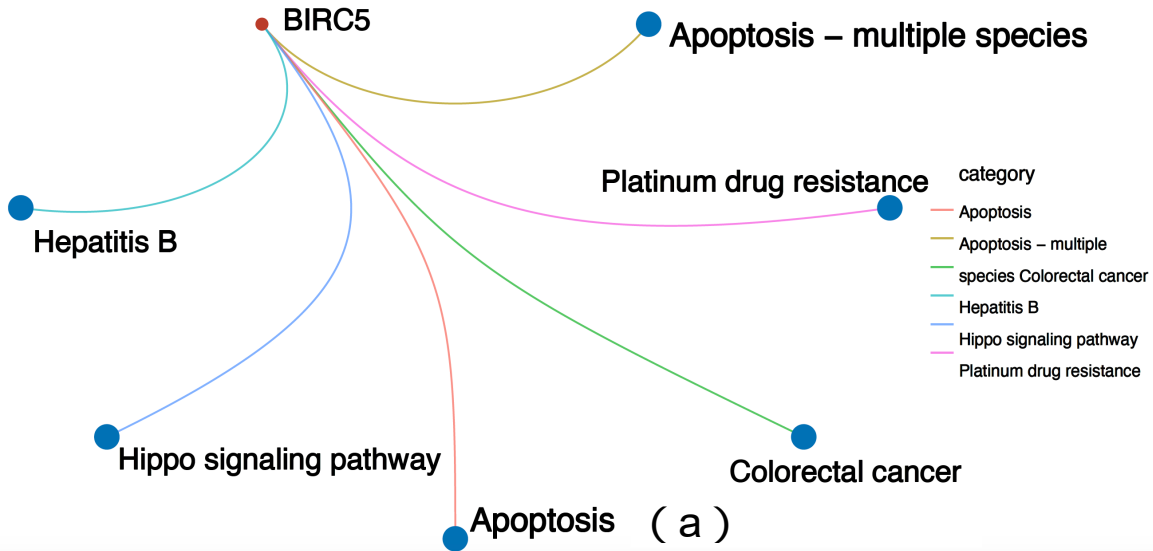

Supplement: Supplementary file 1 [file ijms-24-14663-s001.zip › Figure S4.pdf]
